# Assessment of collagen deposits after implant of fascia lata and fat in the vocal folds of rabbits: histomorphometric study

**DOI:** 10.1016/S1808-8694(15)31251-9

**Published:** 2015-10-20

**Authors:** Christiano de Giacomo Carneiro, Luiz Ubirajara Sennes, Paulo Hilário Nascimento Saldiva, Domingos Hiroshi Tsuji, João Aragão Ximenes Filho

**Affiliations:** 1 Ph.D. in Otorhinolaryngology, FMUSP, Otorhinolaryngologist; 2 Full Professor in Otorhinolaryngology, FMUSP, Associate Professor; 3 Faculty Professor, Department of Clinical Pathology, FMUSP; 4 Ph.D. in Medicine, Medical School, USP (Invites Professor, Post-graduation in Surgery, Medical School, UFC)

**Keywords:** larynx/anatomy & histology, rabbits/surgery, transplatatrion, autologous/side effects

## Abstract

Several materials have been injected or introduced in the vocal folds in attempt of solving the glottic insufficiency. However, few studies have evaluated the cicatricial process due to the implantation of these materials.

**Aim:**

The objective of this research was to evaluate the concentration of collagen after microsurgery graft of muscular fascia and fat in the vocal folds of rabbit.

**Study design:**

experimental.

**Material and Method:**

Nineteen rabbits were submitted to the graft insert in the right vocal fold, being nine of fascia and ten of fat. The left vocal fold was submitted to the same process, except for the insertion of fat or fascial graft. The rabbits were sacrificed after 90 and 180 days. The collagen was analyzed through the method of the Picrosirius-polarization using the Image Pro Plus software.

**Results:**

There was prevalence of the collagen in all grafted groups when compared with the group control. The concentration of the collagen found in the rabbits submitted to fat graft was significantly larger when compared to the concentration of the rabbits submitted to graft of muscular fascia, either with 90 as with 180 days.

**Conclusion:**

The fat and muscular fascia implantation in the vocal folds of rabbit promoted production of collagen, being more intense with fat.

## INTRODUCTION

Human voice's adaptability allows variations of intensity, pitch and quality, enabling expression of emotions and thoughts^1^. Glottic incompetence or failure are among the most important alterations that challenge laryngologists. This condition leads to dysphonia and may be caused by mobility alterations, scars, atrophies or vocal fold bowing^2^. Among such causes, scar - or fibrosis - may be the one most featured by negative social impact and lack of effective treatment^3^.

Collagen is the main element of scarring or fibrotic tissue, which, after tissue damage, begins synthesis on the third day^4^ and reaches its highest peak between 3 and 6 weeks, followed by a remodeling phase. Type III collagen is initially synthesized and deposited, being replaced by type I, while maturation of the healing tissue occurs^5^.

Several synthetic or biological grafts were used in the attempt of repairing the body-cover relationship of vocal fold, in case of alteration of lamina propria. Teflon injection, much adopted in the past, is no longer used due to risk of immunological reactions, granuloma formation and extrusion^6^; currently, it is used only for treatments of cases with limited life-expectations^7^. Limited longevity of gel foam restrains its use as conclusive treatment^8^, although its applicability remains while an accurate modality is defined. More recently, collagen – initially the bovine type, then the autologous^9^, followed by the homologous^10^ – was also used. High cost and high re-absorption rate restrained its use.

Autologous grafts, such as fat and muscular fascia, showed promising results, while safe and minimal risks of unwanted reactions to the graft stimulated its use. Fat is highly bioavailable^11^, besides presenting similar viscosity to the superficial layer of the lamina propria, suggesting that its consistency may be almost optimal for vocal fold injection^12^. Longevity of fat grafts is questionable and dependent on many factors, such as: size and grade of graft manipulation, anatomical region of collection and anatomical region to be implanted^13^.

Muscular fascia is considered one of the surgical autologous possibilities to be used in laryngology. Formerly, it was largely used in reparative plastic surgeries. Muscular fascia may be used due to several benefits: it is a delicate tissue, although it is tension-resistant; it is composed by fibroblasts (collagen) and is bioavailable. The main disadvantage, though, is related to its higher adjustment level to fill in bidimensional spaces, such as in cosmetic surgeries, but not three-dimensional ones, such as in vocal folds^13^.

As to materials used, the literature brings many studies14., 15., 16., 17., 18. regarding graft distribution, inflammatory processes, cell type and quantification; however, studies emphasizing the analysis of repairing tissue produced after graft of these materials are rare^19^.

The objective of this research study was to describe, compare and quantify the collagen resultant from muscular fascia and vocal fold grafting in the vocal fold of rabbits, as well as to compare them to non-grafted vocal folds, using the picrosirius-polarization method.

## MATERIAL AND METHODS

### Material

This study was approved by the Ethics Committee for the Analysis of Research Projects (CAPPesq) of Hospital das Clínicas, Medical School - University of Sao Paulo (research study 200/02).

Nineteen New Zealand healthy albino male rabbits, weighing between 2.5 and 3.5 kg (5.51 and 7.72 pounds) were used in this study and divided into 4 (four) groups. In the first two groups (F1 and F2), the rabbits were submitted to fascia lata graft insertion on the right vocal fold. Rabbits of other groups (G1 and G2) were submitted to fat autologous en bloc implant, also on the right vocal fold. Among all rabbits, the control group was composed by the left vocal fold, which was submitted to the same procedure, except for graft placement. All operated animals were kept alive, daily assisted and controlled in their scarring process. They were all sacrificed by the same method of endogenous injection with 2.0ml of 20% KCl, and:

• After 90 days of surgery, the right vocal folds of the sacrificed animals formed the Fascia (F1) and the Fat (G1) groups and the left vocal folds formed the Control group (C1);
• After 180 days of surgery, the right vocal folds of the sacrificed animals formed the Fascia (F2) and the Fat (G2) groups and the left vocal folds formed the Control group (C2).

## Methods

### Surgical technique

General anesthesia was performed using Xylazine (5 mg/Kg) associated with Ketamine (50 mg/Kg) in animals, intramuscularly. They were maintained with spontaneous ventilation, through laryngeal incision, without tracheal cannula and fixed by their paws. The rabbits were submitted to median line incision, from the upper margin of thyroidal cartilage up to the lower border of cricoid cartilage, using a 15 scalpel, subcutaneously, allowing exposure of thyroid and cricoid cartilages. Fat was collected for posterior insertion through incision of cervical region to access larynx. Muscular fascia was obtained after skin incision of the anterior and lateral regions of thigh, followed by homeostatic control and light dissection (10 × 10mm).

Cricothyroid membrane was incised in the midline with a 15 scalpel, allowing subglottic visualization. Previously opened thyroid cartilage allowed vocal fold exposure. Both vocal folds were submitted to a 0.3mm incision in their upper margin, followed by thorough mucous detachment along the medial margin.

The procedure was performed using a 3s Hollemback probe – the same used in dental maneuvers, enabling confection of a pouch to receive the graft. Caution was necessary to avoid penetration of the thyroarytenoid muscle. Graft (muscular fascia or fat measured by pachymetry), of approximately 3mm diameter and 1mm height, was implanted in the pouch between musculature and the mucosa of the right vocal fold. The left vocal fold did not receive a graft.

Rabbits received antibiotic therapy with 300000 UI procaine benzylpenicillin, UI potassium benzylpenicillin, diluted in 3ml distilled water, and 0.4 ml per dosage was applied, intramuscularly. The first dosage was injected during the procedure, while the others on a 24-hour basis.

### Histological and morphometric study

After sacrificing the animal, the larynx was removed through cervical incision, followed by removal of median fragment of the right and left vocal folds. All pieces measuring around 3 × 5mm were fixed in 10% formalin and sent for: dehydration with 95% ethylic alcohol, cleared in xylol, impregnated by paraffin at 60°C stove, embedded and then sectioned by a 3μm-microtome. Sections stained by the picrosirius-polarization method were used to view and analyze the collagen fibers. This method consists of Sirius red diluted in saturated aqueous picric acid. Lamina analysis was performed under light microscopy using optical polarization.

The four groups were submitted to descriptive analysis with focus on collagen, on Types I and III classified according to birefringence, as well as on the presence of graft and its state in relation to adjacent muscular structure. In each group, left vocal fold samples in which dissection was performed without graft implantation were analyzed.

A morphometric study was also carried out - including collagen fiber quantification through a digital imaging system of analysis using Image Pro Plus version 4 software -, in which collagen fibers concentration per measured area was determined. Samples of both vocal folds were analyzed to have collagen concentration values.

### Statistical analysis

A Kolmogorov-Smirnov normality test was performed to show normal distribution. For groups’ comparison, T Student test was applied.

## RESULTS

Microscopic analysis of vocal folds stained by the picrosirius-polarization method revealed differences regarding distribution of scarring collagen. Fascia lata of groups F1 and F2 presented spindle aspects (Photos 1 and 2), while fat (G1 and G2) presented diffuse aspect, forming focuses encapsulated by thick collagen (Photos 3 and 4). Type I collagen is prevalent (greater birefringence) in all groups, although in G1 and G2, type III collagen is better identified when compared with fascia muscular group, independently of time.

Table 1 summarizes the average concentration of collagen and its standard bias in all studied groups, regarding both vocal folds. Statistical analysis of collagen concentration per area demonstrated an increase of collagen in all grafted groups when compared with control groups (p < 0.001).

**Table 1:** Concentration of collagen in the groups studied.

Groups		G1		G2		F1		F2
Site	PVD	PVE	PVD	PVE	PVD	PVE	PVD	PVE
Average	0,363	0,066	0,273	0,061	0,185	0,074	0,174	0,067
Standard deviation	0,021	0,004	0,018	0,001	0,007	0,005	0,007	0,004
deviation								

PVD: right vocal fold; PVE: left vocal fold

**Graph 1:**
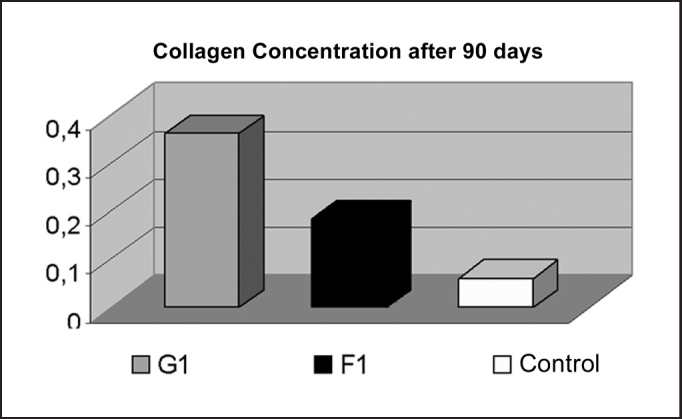
Comparison of Collagen Concentration after 90 days, demonstrating higher concentration in the fat grafting group (p < 0.001).

**Graph 2:**
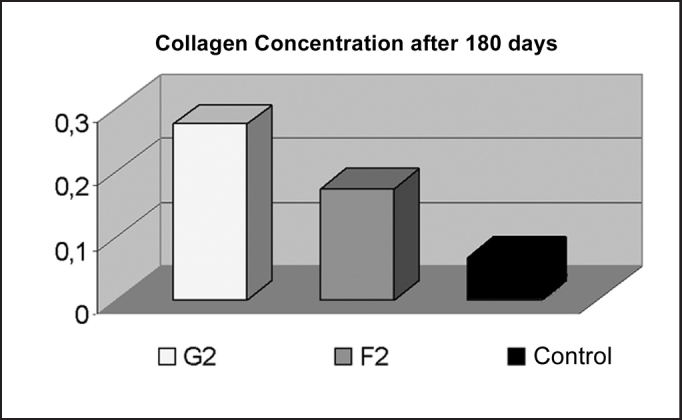
Comparison of Collagen Concentration after 180 days, demonstrating higher concentration in the fat grafting group (p < 0.001).

A higher concentration of collagen was observed in the fat group when compared to the fascia group, both in the 90-day analysis (p < 0.001) and the 180-day analysis (p < 0.001), respectively showed in Graph 1, Graph 2.

**Graph 3:**
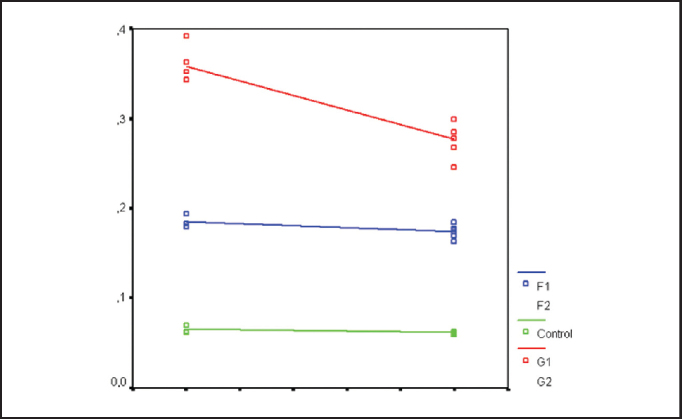
Comparison of Collagen Concentration after 90 and 180 days, demonstrating statistically significant reduction on fat grafted rabbits only.

Comparing the above groups, we observed statistically significant reduction of collagen concentration (p < 0.001), of approximately 25% in this period. In the fascia group, however, there was no difference among the 90 and 180-day groups (p = 0.08), with variation of about 6% of collagen concentration in the period. In the control group, no differences were observed between the period of 90 and 180 days (Graph 3).

## DISCUSSION

Picrosirius-polarization method has significantly contributed for identification and understanding of collagen and its functions. Method specificity is based on the presence of basic aminoacids in collagen molecules, which strongly react with the acid dye (Sirius red). This reaction enhances collagen normal birefringence composed by aggregated molecules. Differentiation of collagen types is also possible, while type I is strongly birefringent with thick yellow fibers; type III has poor refringence and is greenish, and type II is even less refringent^20^^,^^21^.

Presence of abnormal arrangement of collagen fibers leads to development of scar or fibrosis^3^. In our study, disarrangement of collagen was frequent when compared with collagen distribution of vocal muscle of the rabbit in all groups. This alteration was strongly identified in groups submitted to fat graft, characterized by disarrangement in blocks layered by thick collagen. In F1 and F2 groups (fascia graft), distribution is spindle form and more compact.

Literature provides a significant amount of articles emphasizing the inflammatory process resultant from vocal folds grafting, including qualification and cell typing, in an attempt of repairing pre-existent defects or those voluntarily caused by the lamina propria^22^^,^^23^. However, studies with the purpose of analyzing the scarring process caused by manipulation and grafts are rare. Thibealt et al. (2002)^24^, in an histology and rheological study of vocal fold scarring produced by lamina propria followed by surgical manipulation, found lower collagen density associated with tissue disorganization. The present study covers an evaluation of the scarring process when compared to vocal muscle, in which higher collagen density and tissue disorganization are found.

It is believed that low metabolic rate and ultra-structure formed mainly by fibrocytes and collagen extracellular matrix are the main actors in the longevity of fascial graft of the vocal folds^16^. Reijonen et al. (2001)^15^, after injection of fascia lata in paralyzed vocal fold of 9 dogs, identified that the muscular fascia did not foster intense inflammatory reaction, which was verified up to 12 months after injection. In a clinical study with patients previously submitted to fat injection, Duke et al. (2001)^18^ obtained 95% improvement in the acoustic analysis of voice standards.

Rabbit vocal fold is similar to human vocal fold, which demonstrates division by layers and presence of cells from the collagen extracellular matrix. A limitation for the present study is the fact that rabbit vocal folds do not present the same mechanical vibration forces as the human vocal fold. It has been reported that these forces may stimulate collagen turnover with consequent alteration in the fibers deposition^25^. Notwithstanding, these findings suggest that, even though grafting promotes a higher collagen deposition in the scarring process, this is more exuberant with fat than with fascia. However, thorough studies on the rheological peculiarities of vocal folds grafted with fat and fascia are necessary to better define the optimal type of graft.

## CONCLUSION

The study leads to the conclusion that fat and fascia lata grafting in rabbit vocal fold promoted greater collagen deposition than the control group, being more exuberant in fat insertion. Distribution of collagenic fibers was altered after fascia and fat graft implantation, the latter presenting greater changes.
